# Stable Isotopes Reveal Trophic Partitioning and Trophic Plasticity of a Larval Amphibian Guild

**DOI:** 10.1371/journal.pone.0130897

**Published:** 2015-06-19

**Authors:** Rosa Arribas, Carmen Díaz-Paniagua, Stephane Caut, Ivan Gomez-Mestre

**Affiliations:** 1 Ecology, Evolution, and Development Group, Department of Wetland Ecology, Doñana Biological Station, CSIC, Seville, Spain; 2 Department of Ethology and Conservation of Biodiversity, Doñana Biological Station, CSIC, Seville, Spain; Stockholm University, SWEDEN

## Abstract

Temporary ponds are highly variable systems where resource availability and community structure change extensively over time, and consequently the food web is highly dynamic. Amphibians play a critical role both as consumers and prey in aquatic communities and yet there is still little information on the trophic status of most amphibians. More importantly, little is known about the extent to which they can alter their trophic ecology in response to changing conditions. We experimentally investigated the effects of increased amphibian density, presence of intraguild competitors, and presence of native and invasive predators (either free or caged) on the trophic status of a Mediterranean amphibian guild, using stable isotopes. We observed variations in *δ* ^13^C and *δ* ^15^N isotopic values among amphibian species and treatments and differences in their food sources. Macrophytes were the most important food resource for spadefoot toad tadpoles (*Pelobates cultripes*) and relatively important for all anurans within the guild. High density and presence of *P*. *cultripes* tadpoles markedly reduced macrophyte biomass, forcing tadpoles to increase their feeding on detritus, algae and zooplankton, resulting in lower *δ* ^13^C values. Native dytiscid predators only changed the isotopic signature of newts whereas invasive red swamp crayfish had an enormous impact on environmental conditions and greatly affected the isotopic values of amphibians. Crayfish forced tadpoles to increase detritus ingestion or other resources depleted in *δ* ^13^C. We found that the opportunistic amphibian feeding was greatly conditioned by intra- and interspecific competition whereas non-consumptive predator effects were negligible. Determining the trophic plasticity of amphibians can help us understand natural and anthropogenic changes in aquatic ecosystems and assess amphibians’ ability to adjust to different environmental conditions.

## Introduction

Studying the diet of individuals within a community provides key ecological insight about food web structure essential to understand trophic relationships, ecological roles, and niche partitioning [1–3]. This information is of utmost importance to understand the structure of natural communities and to predict the consequences of ecological disturbances such as habitat modification, global climate change, or introduction of invasive species [4,5]. Interactions among individuals and with the environment modulate variations in community diversity, structure, and dynamics [6], and also influence niche width of the interacting species at an ecological and evolutionary scale [7,8]. Trophic niche partitioning within aquatic communities has been studied through the analysis of functional groups in macroinvertebrates [9,10] and fish [11], and more recently via determination of isotopic signatures [12–16].

Amphibian larvae are pivotal agents in structuring aquatic communities, acting as primary consumers, as prey for both vertebrate and invertebrate predators, but also as predators themselves [17]. Thus, amphibian larvae can have profound effects on nutrient availability, zooplankton composition, and macrophyte biomass [18]. Surprisingly, despite their potential cascading consequences for the rest of the community, relatively little work on their trophic ecology has been conducted (but see [19–22]). Tadpoles of many species are considered herbivores that also feed on detritus, bacteria, plankton or fungi [23], although our knowledge about what tadpoles really eat has been put into question [20]. The diet of larval urodeles has been studied for particular species, all of which are carnivorous and can have a great impact on aquatic communities (see review in [17]). In contrast, the diet of anuran larvae has seldom been described and actual direct information about diet composition has only been described for a limited number of species [20]. Instead, their trophic niche has rather been inferred from their mouth morphology or their most frequent position in their aquatic habitats [17,20,24]. Tadpole morphology shows a wide spectrum of feeding specializations, which allows classifying them into functional groups, ranging from typical herbivorous feeding on phytoplankton to cannibalistic species, including species with endotrophic larvae that entirely rely on yolk [17,20].

Despite general predictions about the trophic status or ecological specialization at the species level according to behavior, preferred habitats, and structure of the mouthparts, trophic ecology of amphibian larvae is likely to be complex and contingent upon the environmental conditions experienced. Most amphibians breed in highly dynamic aquatic systems, like temporary ponds or flooded river banks which show extensive variation in space and time regarding availability of potential resources for amphibian larvae [25]. To complicate things further, trophic ecology of tadpoles may not only vary due to high environmental dynamism, but also because tadpoles respond plastically to such environmental fluctuations by altering their own behavior and morphology [26,27]. Their trophic status may consequently be rather plastic and change in response to interactions with competitors and predators. In amphibian larvae, competitors and predators induce changes in morphological traits that could have direct implications in their diet, such as gut length, oral disc size, beak width, and tooth rows [28]. Tadpoles also reduce their activity rate or increase the use of refugia in the presence of predators [26,29], which will cause changes in their diets if they start feeding in different parts of the water column, shifting from a macrophyte-based diet to a periphyton- or a phytoplankton-based one. However, recently introduced predators that did not share a joint evolutionary history with tadpoles do not trigger such behavioral responses [30,31]. Despite the great potential importance for aquatic systems of plastic changes in the trophic ecology of amphibian larvae [32–34], there is still a lack of information regarding how ecological factors may alter diets within local amphibian guilds [17]. Traditional methods to determine diet composition such as gut contents, fecal analysis, or foraging observations only give information about the most recent ingestion, although they can be very informative if sampling is extensive over time and across age and size classes. However, stable isotope analysis can further provide an account of the diet that the organisms have had over a longer period of time and may help revealing the specific resources used by consumers [35]. Stable isotopes have been recently used in amphibian larvae, as for example in assessing ontogenetic diet variations or their implications in food webs [36,37]. Carbon isotopes (*δ* ^13^C) identify major energy sources, and nitrogen isotopes (*δ* ^15^N) indicate trophic position within a food web [13].

The present study constitutes a follow up to a comprehensive mesocosm experiment designed to assess the effect of larval amphibians on physico-chemical and biotic characteristics of ponds under different ecological conditions [18]. We experimentally manipulated amphibian larval density and the presence or absence of a large tadpole species that acts as a strong competitor. Moreover, we also manipulated the presence/absence of native and invasive predators, kept either caged or freely roaming to distinguish their non-consumptive effects on the trophic status of amphibians. Free predators would exert both direct density-dependent effects and non-consumptive effects whereas caged predators would only have the potential to have non-consumptive effects, mostly via induction of altered behavior in the tadpoles. We reported that amphibian larvae at high density, as well as invasive crayfish had marked effects on the aquatic environment, altering water physico-chemistry, plant biomass, and zooplankton abundance [18]. At high larval density, amphibians decreased macrophyte biomass, reduced zooplankton diversity, caused increased water turbidity, and increased the presence of nutrients in the water [18]. The large spadefoot toad tadpoles (*Pelobates cultripes*) were the main contributors to these changes. Free-roaming invasive crayfish (*Procambarus clarkii*) caused analogous effects to that of high amphibian density, and they also caused greater amphibian mortality (79.6%) than native dragonflies did (65%) [18]. These changes in pond characteristics would be expected to imply substantial changes in the abundance of trophic resources available to the amphibian larvae, consequently causing shifts in their trophic status.

Our aim in this study was to assess the trophic plasticity of a guild of amphibian larvae under different ecological conditions. For this purpose, we used stable isotopes to analyze how the changes in pond characteristics due to amphibian density and predator presence would be reflected in diet shifts of the amphibian species studied. We hypothesized that amphibian larvae would alter their carbon source and present a lower trophic level (lower *δ* ^15^N values) with increased larval density and when faced with strong competitors like *P*. *cultripes* tadpoles. We expected lower *δ* ^15^N values due to reduced availability of resources and an increase in the relative consumption of detritus. We also expected shifts in the carbon source of amphibian larvae and higher *δ* ^15^N values in the presence of native predators, because they reduced amphibian density thus relaxing inter- and intraspecific competition. We expected this to result in the maintenance of a greater variety of resources available for amphibians. On the other hand, we expected lower *δ* ^15^N values in amphibian larvae exposed to caged native predators due to induced lower foraging activity. However, we expected no such changes in trophic status in the presence of caged invasive predators due to lack of innate recognition. Nonetheless, the dramatic impoverishment of the environmental conditions produced by free-roaming crayfish [18] would force amphibians to seek alternative carbon sources.

## Materials and Methods

### Ethics statement

Amphibian larvae, dytiscid beetle larvae, and red swamp crayfish were collected within the Biological Reserve at Doñana National Park with collecting permits granted by Consejería de Medio Ambiente from Junta de Andalucía. The experimental procedures and euthanasia of tadpoles were conducted at Reserva Biológica de Doñana, CSIC, following protocols approved by the Institutional Animal Care and Use Committee (IACUC) at CSIC (Ref. CGL2012-40044).

### Study system

This study was carried out in Doñana National Park, located in the southwest of Spain on the right bank of the Guadalquivir River mouth (37°00’N, 6°38’W). The climate of this area is Mediterranean with Atlantic influence, with hot and dry summers and mild winters. One half of the park is a sandy area with an important temporary pond network and the other half is an extensive seasonal marsh (more details in [18]). The amphibian community is composed of eleven species (eight anurans and three urodeles), of which the most abundant are those that use temporary ponds as breeding habitats. We included in our study the larvae of six of the most abundant species (five anurans and one urodele) to have a good representation of the community of the area. The anurans include in their diet a variable proportion of detritus, algae and phanerogams. However, these anurans range from mainly bottom-dwelling tadpoles feeding on detritus and periphyton (the natterjack toad, *Bufo calamita;* the Iberian painted frog, *Discoglossus galganoi;* and the Iberian green frog, *Pelophylax perezi*) to water column feeders, mainly on macrophytes (the western spadefoot toad, *Pelobates cultripes*) or filtering algae (the Mediterranean treefrog, *Hyla meridionalis*) [38]. The larvae of the urodele, the pigmy marbled newt (*Triturus pygmaeus*), mainly feed on planktonic crustaceans [39].

### Experimental array

We established an experimental mesocosms array at the central area of the park consisting of 7 treatments (see Table 1 for an overview): 1) Low amphibian density **(** *Low* **)**: presence of low density of larvae of six amphibian species; 2) High density of amphibian larvae (*High* **)**: three-fold low density number; 3) Absence of *P*. *cultripes* **(** *No Pc* **)**: same as in the Low treatment but excluding *Pelobates cultripes*, the largest species with the longest developmental period in the area; 4) Caged native predator (*NatC*): Low density of amphibian larvae exposed to a single caged *Dytiscus* larva. Caged predators provide chemical cues but do not reduce amphibian survival by predation; 5) Free native predator (*NatF*): Low density of amphibian larvae exposed to a single free ranging *Dytiscus* larva, which can provide chemical cues and reduce amphibian survival; 6) Caged invasive predator (*InvC*): Low density of amphibian larvae exposed to a caged red swamp crayfish; 7) Free invasive predator (*InvF*): Low density of amphibian larvae exposed to a single free ranging red swamp crayfish; and 8) Control (*No Amph*): absence of amphibians and predators. We considered low density to be 3 individuals per tank for *P*. *cultripes*, *P*. *perezi*, *D*. *galganoi*, and *T*. *pygmaeus*, 10 individuals per tank for *H*. *meridionalis*, and 15 individuals per tank for *B*. *calamita*. The high-density treatment increased three-fold each species’ density. Tadpole densities were chosen according to the natural range of abundances in the natural ponds in the area [40].

**Table 1:** Overview of the experimental treatments specifying name, acronym, number of replicates and details of each treatment.

Treatment name	Acronym	Replicates	Details
Low amphibian density	*Low*	12	Presence of 6 species of amphibian larvae at low density
High amphibian density	*High*	12	3-times the density of amphibian larvae in the Low treatment
Absence of *P*. *cultripes*	*No Pc*	12	Same as in low density but without the presence of one species (*Pelobates cultripes*)
Caged native predator	*NatC*	12	Low density of amphibian larvae together with a caged *Dytiscus* larva
Free native predator	*NatF*	12	Low density of amphibian larvae together with a free *Dytiscus* larva
Caged invasive predator	*InvC*	12	Low density of amphibian larvae together with a caged red swamp crayfish
Free invasive predator	*InvF*	12	Low density of amphibian larvae together with a free-roaming red swamp crayfish
Control	*No Amph*	12	No amphibians and no predators

Each treatment was replicated 12 times for a total of 96 tanks distributed in 12 randomized blocks.

The experiment lasted for 10 weeks between March 24th and June 2nd 2011. Mesocosms were 500-L plastic tanks, with an upper diameter of 120 cm, and covered with tightly fitting lids of fiberglass window screening. Tanks were filled three weeks prior to the onset of the experiment as described in Arribas *et al*. [18], with a combination of sand and pond sediment from nine different temporary ponds prior to fill them with well water, replicating the natural conditions of ponds in Doñana. In addition, we added pond water as inoculum for zoo- and phytoplankton, and planted in each tank the same amount of three species of abundant aquatic macrophytes of the area (*Myriophyllum alterniflorum*, *Ranunculus peltatus*, and *Callitriche obtusangula)* to provide greater spatial complexity and nutrient availability for the aquatic community.

The six species of amphibian larvae, crayfish, and dytiscid beetle larvae were collected by dip-netting in different temporary ponds within the park. We introduced them in the experimental tanks following the natural breeding phenology of the species [41] and according to the rainfall patterns of the season. Thus, we first introduced *P*. *cultripes* and *B*. *calamita*, then *H*. *meridionalis* and *T*. *pygmaeus*, then *D*. *galganoi*, and lastly *P*. *perezi* (see [18]). Amphibian larvae were similar in initial size and were randomly distributed across tanks (see S1–S4 Tables). Predator cages consisted of lidded 1L plastic buckets with small holes drilled at the bottom to allow predator cues to diffuse throughout the tank. Depending on the treatment, we kept these cages empty or included either a dytiscid larvae in the native predator treatment or a red swamp crayfish in the invasive predator treatment. Two amphibian larvae randomly taken from the six species of the study were introduced in the cages with predators to feed them during the experiment. We checked the tanks every day to record metamorphosis date of individuals of each species (i.e. Gosner stage 42, [42]) until the end of the experiment, when tanks in some treatments had been depleted of macrophytes and when natural ponds had begun to dry up. At that point, we removed every larvae or metamorph remaining in the tanks. At the end of the experiment, we observed steep reductions in amphibian survival in treatments where invasive and native predators were free, as well as under high amphibian density.

### Isotopic analysis

We could only take samples for isotopic analyses from four out of the six species of amphibians, because there were no survivors of *B*. *calamita* and very few survivors of *D*. *galganoi* at the end of the experiment. We collected one or two individuals from each species and tank, when available, and euthanized them by immersion in MS-222. The anesthetic MS-222 is commonly used for euthanizing aquatic organisms and no effects have been reported on stable isotope measurements (e.g., [43,44]). We collected individuals as they metamorphosed and larvae from tanks from which we had not yet recovered metamorphic individuals. Metamorphs and larvae of each species collected were kept at -20 °C until processed for isotopic analysis. Most individuals processed of *H*. *meridionalis* had already metamorphosed by the end of the experiment, whereas most individuals of *P*. *perezi* were still in the larval stage (see Table 2), as is usually the case in natural ponds.

**Table 2:** Number of individuals (metamorphs and larvae) used for isotopic analyses in each species and treatment.

Species	*Hyla meridionalis*	*Pelobates cultripes*		*Pelophylax perezi*		*Triturus pygmaeus*	
	Metamorphs	Larvae	Metamorphs	Larvae	Metamorphs	Larvae	Metamorphs
**Treatment**							
**Low**	10	7	9	7	1	7	2
**High**	1 (+ 5 larvae)	10	8	9	2	9	1
**No Pc**	11	-	-	9	2	12	2
**NatCaged**	12	4	10	8	3	10	1
**NatFree**	9	4	12	7	3	7	2
**InvCaged**	11	5	11	8	3	10	3
**InvFree**	7	2	5	7	0	0	0
TOTAL	66	87		69		66	

We determined carbon and nitrogen isotopic values (*δ* ^13^C and *δ* ^15^N) from muscle samples of amphibians, dissecting tail muscle from tadpoles and newts, and leg muscle from anuran metamorphs (n = 288, but see Table 2 for specific number of individuals processed per species and development stage). Each sample was kept in a separate labeled eppendorf tube, oven-dried at 50°C to constant mass and ground to a fine and homogeneous powder with mortar and pestle.

All possible resources for amphibians in the mesocosms were collected one month after the experiment started from six additional tanks randomly chosen out of the twelve tanks with the same conditions but containing neither amphibians nor crayfish or dytiscids (*No amph*) [18]. In this study, we considered detritus, algae, zooplankton, three species of macrophytes, and one species of charophytes that emerged from the sediment, as possible carbon sources for consumers. We considered these food sources appropriate to perform the isotopic analysis as we have previous and detailed information on the diet of the species studied. The anuran species studied are mainly herbivorous, feeding on algae, macrophytes, fungi and detritus with a small fraction of the diet contributed by animal matter and bacteria [38]. In contrast, larval newts are carnivorous, feeding mainly on zooplankton [39], although other particles may accidentally be ingested. For this reason, we included all seven food items to calculate the relative contribution of each source to the diet of tadpoles, but only included zooplankton and detritus as possible food sources for newts.

For detritus samples, we carefully collected 4 cm^2^ of the upper 1 cm sediment layer in each additional tank. In order to collect the finer top particles, we centrifuged the sample for 5 minutes at 1100 G in 15 mL conic Falcon tubes and preserved the first 3 mm of the pellet for analyses. We then divided the sample in two, and treated one of them sequentially with HCl 0.1M and 1M to remove any carbonates [45]. We cleaned this sample with several centrifugations with distilled water until the supernatant was clear. The isotopic values for *δ* ^13^C were obtained after treating them with HCl, and for *δ* ^15^N were obtained from the other untreated part of the sample. To collect zooplankton, we filtered 5 L of water from each of the additional tanks through a 100μm net and kept the individuals in the filter with dechlorinated tap water. We then took each water sample, centrifuged them, and kept only the portion of the water column containing swimming zooplankton. For algae, we filtered two extra liters of water from these tanks through a smaller mesh size (60 μm), placed the water in a glass container and allowed it to evaporate in an oven at 50°C, and then scraped the remains from the walls and the bottom for isotopic analysis of all the microalgae found there. All samples of potential resources were dried up in the oven at 50°C to constant mass prior to isotopic analysis and then ground to a fine and homogeneous powder.

We weighed 0.3 ± 0.01 (mean ± SD) mg of amphibian muscle, 2.16 ± 0.05 mg of algae, 1.02 ± 0.14 mg of zooplankton, 10.6 ± 0.73 mg of detritus, and 1.66 ± 0.04 mg of macrophytes and placed them into tin capsules for *δ* ^13^C and *δ* ^15^N determinations. These weights depend on nitrogen concentration of the samples as well as on the resolution of the spectrometer. Isotopic analyses were carried out at the Laboratory of Stable Isotopes at Doñana Biological Station. All samples were combusted at 1,020°C using a continuous flow isotope-ratio mass spectrometry system by means of Flash HT *Plus* elemental analyzer coupled to a Delta-V Advantage isotope ratio mass spectrometer via a CONFLO IV interface (Thermo Fisher Scientific, Bremen, Germany). Stable isotope ratios are expressed in the standard *δ*-notation (‰) relative to Vienna Pee Dee Belemnite (*δ* ^13^C) and atmospheric N2 (*δ* ^15^N), using the equation *δ* ^13^C or *δ* ^15^N = ((R_sample_ ⁄R_standard_)– 1) x 1000, where R is ^13^C/^12^C or ^15^N/^14^N. Replicate assays of laboratory standards routinely inserted within the sampling sequence, and previously calibrated with international standards, indicated analytical measurement errors of ± 0.1‰ and ± 0.2‰ for *δ* ^13^C and *δ* ^15^N, respectively. We calculated the mean and standard deviation for the seven sources included in this study, for *δ* ^13^C and *δ* ^15^N as well as for %C and %N (elemental composition). Post et al. [46] recommended correcting the *δ* ^13^C values for lipid content when the C/N ratio of the tissue is > 3.5 in aquatic animals. Following this approach, we normalized *δ* ^13^C values to account for lipid variation in *δ* ^13^C (mean C/N = 3.51) following the equation in Caut et al. [43], derived for amphibian tadpoles: *δ* ^13^C_normalized_ = *δ* ^13^C_untreated_—1.11 + 0.37 (C/N).

### Statistical Analyses

We performed statistical analysis with SAS 9.2 (SAS-Institute, Cary, NC, USA) and R 3.0.2 software [47]. We evaluated the significance of diet differences between individuals at the tadpole stage and individuals at the juvenile stage by means of two-sample t-tests. We used generalized linear mixed models (GLMM) to test for the effect of experimental treatments, species, or ‘treatment x species’ interactions on the mean values of *δ* ^13^C or *δ* ^15^N for each amphibian species and tank. Values of *δ* ^13^C were log-transformed to meet parametric assumptions whereas *δ* ^15^N values met the assumptions and no transformation was necessary except for the species *P*. *cultripes*, where we ranked the variable (ties method = minimum) prior to analysis with general linear models. We tested for differences in isotopic values among treatments using the GLIMMIX procedure from SAS statistical package [48]. In all models fitted, we first tested the effect of experimental block as a random factor, but finally removed it from the model since it was never significant. Normally distributed variables were modeled with a Gaussian error distribution and an identity link function. Since our experimental design was not fully factorial, we specified multiple contrasts through planned comparisons to test specific hypotheses: we tested for effects of increasing amphibian density with the contrast *Low*-*High*; we tested the effect of the big sized tadpoles *P*. *cultripes* comparing *Low*-*No Pc*; and we tested the effect of predators with that of the amphibian presence at low density (*Low*) and comparing caged and free predators. Given that several of these multiple comparisons (8 comparisons total) were not orthogonal, we corrected the resulting *p*-values to minimize the false discovery rate (FDR, [49,50]). The FDR is a simple and powerful method for controlling type I error when multiple comparisons are carried out, keeping the proportion of those errors low [51].

We checked for differences on the isotopic values and on C and N content of the potential food sources by means of a non-parametrical Kruskal-Wallis test (K-W). The relative isotopic contribution of each food item to a consumer’s diet was calculated using the Bayesian stable isotope mixing model Stable Isotope Analysis in R (SIAR package, [52]). The model calculates the range of all possible sources’ contributions for systems where the number of potential sources can be greater than n + 1, n being the number of isotopes analyzed. Isotopic models typically use the mean *δ* ^13^C and *δ* ^15^N values and standard deviations for each type of diet, corrected for the discrimination factor of the consumer (the increase in consumer isotopic ratio compared to its diet, noted *Δ* ^13^C and *Δ* ^15^N). Concentration dependence or groups were not included in the models. Discrimination factors depend on several sources of variation (e.g., taxa, site, tissue; see review in [53]). Previous laboratory work showed significant relationships between *δ* ^13^C and *δ* ^15^N of diets and the corresponding *Δ* ^13^C and *Δ* ^15^N of the tissues of amphibian tadpole fed on these diets [43]. For both tadpoles and metamorphs, we calculated the diet-dependent discrimination factors corresponding to each potential diet item with the equations *Δ* ^13^C = -0.35 *δ* ^13^C -7.70 and *Δ* ^15^N = 0.53 *δ* ^15^N -0.37 from Caut et al. [43].

## Results

### General isotopic differences among species and treatments

We collected between 7 and 18 individuals per species per treatment (nested within tank), summing up a total of 288 individuals for determination of isotopic values. The number of individuals analyzed for each species is shown in Table 2 (larval and metamorph sizes are described in S1–S4 Tables for each species). Values of *δ* ^13^C and *δ* ^15^N did not differ between tadpole and metamorph stages within species (all t-tests *P*>0.09) and we therefore pooled all samples within species regardless of stage for further analyses.

**Fig 1:**
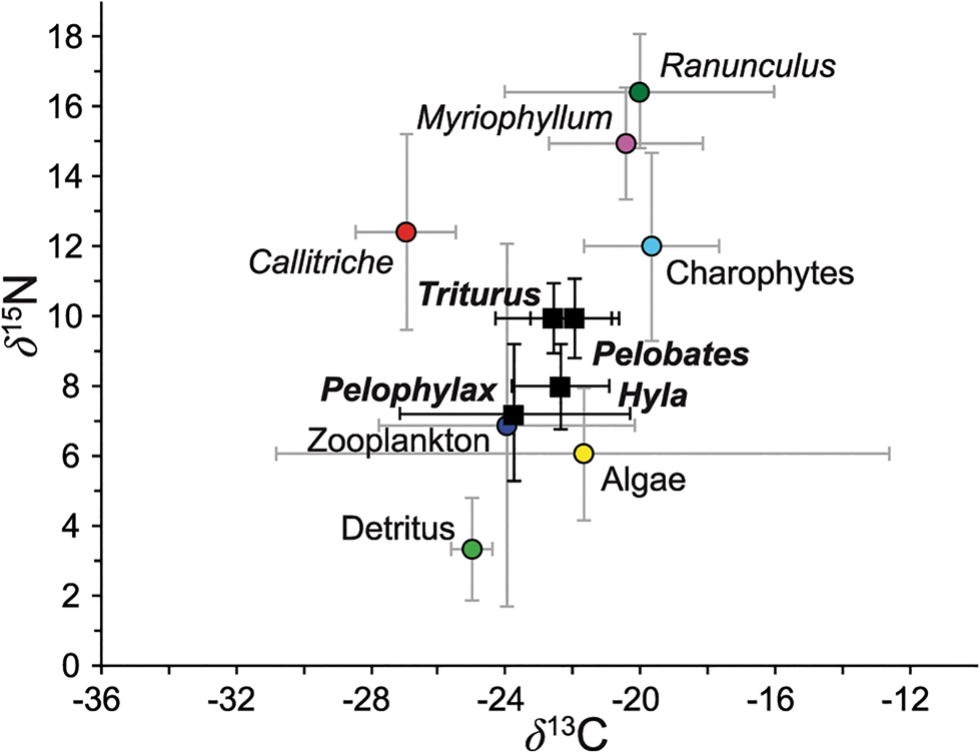
Stable isotope biplot of each amphibian species and sources of the experiment. *δ* ^15^N and *δ* ^13^C values (mean ‰ **±** SD) of the amphibians included in this study (*Hyla meridionalis*, *Pelobates cultripes*, *Pelophylax perezi*, and *Triturus pygmaeus*) pooled across all experimental treatments for a general view of differences in trophic status among species. Resources are also shown as means **±** SD. The discrimination factors were added to the sources and not subtracted to the consumers to allow different discrimination factors to be assigned to different sources.

We found significant species-by-treatment interactions for both isotopes (*δ* ^13^C: F_16,262_ = 3.54; *P<*0.0001; *δ* ^15^N: F_16,262_ = 4.66; *P<*0.0001) indicating that the effect of experimental treatments on the diet varied among species. Muscle *δ* ^13^C and *δ* ^15^N values differed among species (*δ* ^13^C: *F*_3,262_ = 13.33; *P<*0.0001; *δ* ^15^N: F_3,262_ = 81.1; *P<*0.0001) and among treatments (*δ* ^13^C: F_6,262_ = 23.83; *P<*0.0001; *δ* ^15^N: F_6,262_ = 8.07; *P<*0.0001). Pooling across treatments, we found higher *δ* ^15^N values in *P*. *cultripes* and *T*. *pygmaeus*, and the lowest values in *P*. *perezi*. For *δ* ^13^C, we also found the lowest values in *P*. *perezi* (Fig 1).

#### The effect of larval density and species interactions on trophic niche

**Fig 2:**
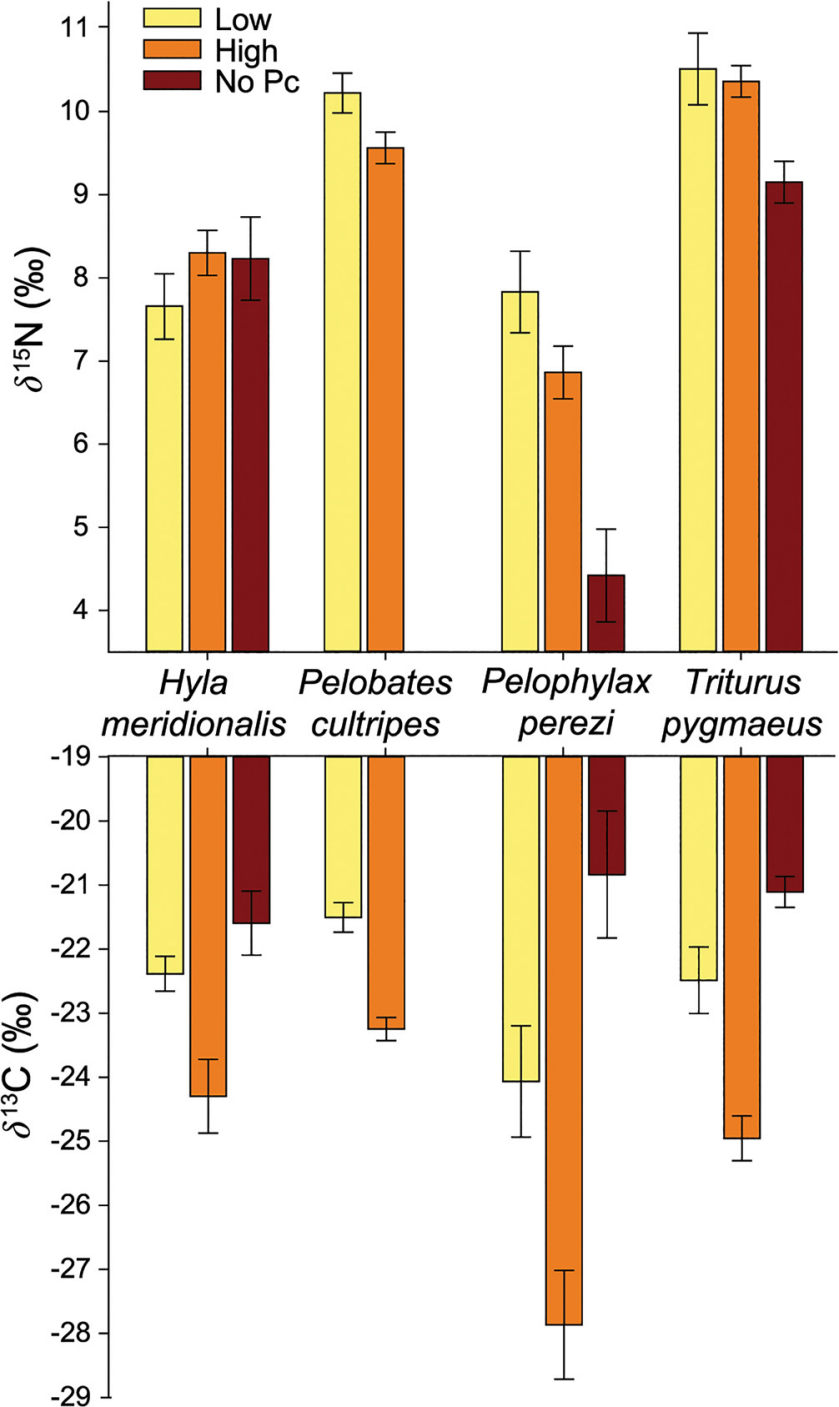
Stable isotopic values for each amphibian species in the density treatments of the experiment. *δ* ^15^N and *δ* ^13^C values (mean ‰ **±** SE) of *H*. *meridionalis*, *P*. *cultripes*, *P*. *perezi*, and *T*. *pygmaeus* muscle in three different density treatments: low density of amphibian larvae (*Low*), a three-fold increase of the density from the low treatment (*High*), and exclusion of the competitive species *P*. *cultripes* (*No Pc*).

The isotopic values of each of the four amphibian species changed between low and high density treatments, especially for *δ* ^13^C. All species showed a decrease in 8–16% of *δ* ^13^C values [(final *δ* ^13^C value-initial *δ* ^13^C)/initial *δ* ^13^C] at high density than at low density (*Low*-*High*, all *P<*0.05, Fig 2 and S5 Table). However, *δ* ^15^N values were not significantly affected by high density (all *P*>0.05). Only *P*. *cultripes* showed a trend towards a decrease in *δ* ^15^N when at high density, although it was marginally non-significant (*Low*-*High*: *F*_1,81_ = 2.98; *P* = 0.088; Fig 2).

The exclusion of *P*. *cultripes* caused differences in *δ* ^13^C values in all other species, with higher *δ* ^13^C values for *P*. *perezi* (*Low*-*No Pc*: F_1,62_ = 28.7; *P<*0.0001), *T*. *pygmaeus* (Low-No Pc: F_1,60_ = 6.97; *P* = 0.01) and *H*. *meridionalis* (*Low*-*No Pc*: F_1,60_ = 7.57; *P* = 0.0078), indicating that their carbon source was different in the absence of spadefoot toads. We found lower *δ* ^15^N values in individuals of *P*. *perezi* and *T*. *pygmaeus* when *P*. *cultripes* larvae were absent (*Low*-*No Pc* contrast; *P*. *perezi*: F_1,62_ = 10.7, *P* = 0.0034; and *T*. *pygmaeus*: F_1,60_ = 11.68; *P* = 0.0011; Fig 2). Instead, we observed no changes in nitrogen values among treatments for *H*. *meridionalis* (*Low*-*NoPc*: F_1,60_ = 0.25; *P* = 0.62).

#### Predator effects: type of predator and lack of non-consumptive effects

**Fig 3:**
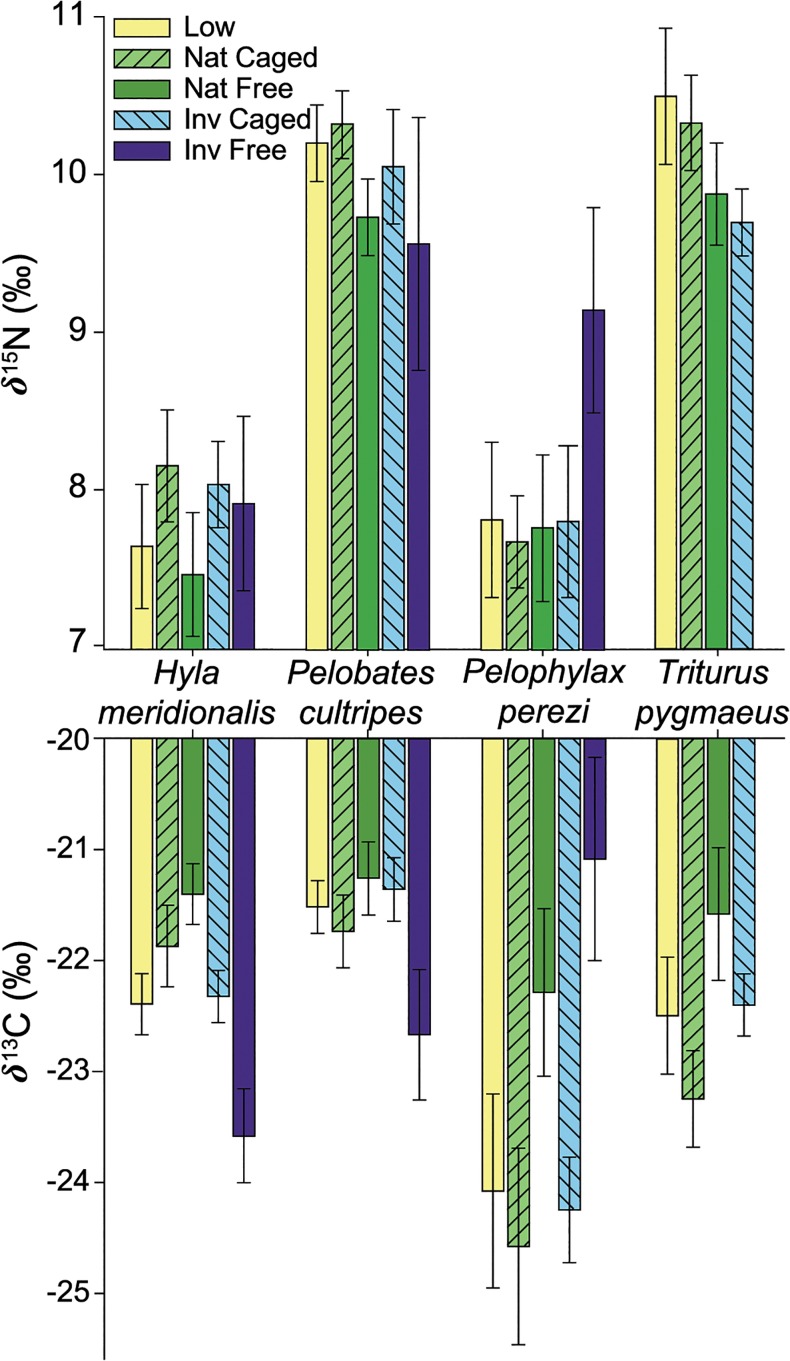
Stable isotopic values for each amphibian species in the presence / absence of predators. *δ* ^15^N and *δ* ^13^C values (mean ‰ ± SE) of *H*. *meridionalis*, *P*. *cultripes*, *P*. *perezi*, and *T*. *pygmaeus* muscle in the presence of native dytiscid larvae (free or caged, *Nat Free* or *Nat Caged*), or in the presence of invasive red swamp crayfish (free or caged, *Inv Free*,or Inv Caged), compared to absence of predators (*Low*).

Predators influenced the isotopic signatures of carbon and nitrogen (Fig 3 and S5 Table). Native predators only exerted significant changes in isotopic values in the newts. We found higher *δ* ^13^C value in *T*. *pygmaeus* when native dytiscid were free in the tanks compared to when they were caged (*NatC*-*NatF*: F_1,60_ = 8.95; *P* = 0.006) although these changes were not significant in the absence of predators (*Low*-*NatF*: F_1,60_ = 2.5; *P* = 0.12). Free crayfish, however, caused the biggest differences in isotopic values. We found significantly lower *δ* ^13^C in the presence of free crayfish compared to the absence of predators for *H*. *meridionalis* (*Low*-*InvF*: F_1,59_ = 4.21; *P* = 0.045) and for *P*. *cultripes* (*Low*-*InvF*: F_1,81_ = 5.24; *P* = 0.025), or when crayfish were caged (*InvC*-InvF: F_1,59_ = 4.97; *P* = 0.044 and F_1,81_ = 6.77; *P* = 0.015 for *Hyla* and *Pelobates* respectively). On the contrary, *P*. *perezi* showed higher *δ* ^13^C in the presence of free crayfish, both compared to the absence of predators (*Low*-*InvF*: F_1,62_ = 4.86; *P* = 0.031) and when the crayfish was caged (*InvC*-*InvF*: F_1,62_ = 6.25; *P* = 0.02).

We detected a non-significant trend towards lower *δ* ^15^N values in tanks with free invasive crayfish for *P*. *perezi* (*Low*-*InvF*; F_1,62_ = 3.09; *P* = 0.08), but crayfish did not affect *H*. *meridionalis* or *P*. *cultripes*. There were no survivors of *T*. *pygmaeus* when free crayfish were free and thus no isotopic analyses could be conducted. However, *δ* ^15^N values for newts were lower when crayfish were caged compared to the absence of predators (*Low*-*InvC*; F_1,60_ = 3.94; *P* = 0.0518, Fig 3).

### Dietary contributions of the different sources to different amphibian larvae

The food sources available for tadpoles in our tanks differed in their *δ* ^13^C (K-W χ^2^_6_ = 28.62, *P*<0.001) and *δ* ^15^N (K-W χ^2^_6_ = 32.45, *P*<0.001) as well as in the percentage of C (K-W χ^2^_6_ = 36.68, *P<*0.001) and N (K-W χ^2^_6_ = 33.54, *P*<0.001) (Table 3). We used a different discrimination factor for each food source in the model input, as *Δ* ^13^C ranged from -0.85 to 2.67 and *Δ* ^15^N from 0.91 to 4.93 (Table 2).

**Table 3:** Stable isotope composition (*δ* ^13^C, *δ* ^15^N), elemental composition (%C, %N) and discrimination factor (*Δ* ^13^C, *Δ* ^15^N) of the potential food sources for amphibian larvae in the experimental arrays of the study.

	Source	*δ* ^13^C ‰	*δ* ^15^N ‰	% C	% N	*Δ* ^13^C	*Δ* ^15^N
	Detritus	-26.6±0.32	2.42±0.68	5.66±0.36	0.55±0.04	1.61±0.11	0.91±0.36
	Algae	-21.51±4.66	4.20±0.75	7.89±1.79	0.44±0.13	-0.17±1.63	1.86±0.4
	Zooplankton	-25.02±1.5	4.68±2.05	23.26±11.5	2.07±1.43	1.06±0.52	2.21±1.09
Macrophytes and Charophytes	*Myriophyllum*	-19.57±1.04	9.95±0.72	44.5±0.74	3.36±0.34	-0.85±0.36	4.93±0.38
	*Callitriche*	-29.62±0.63	8.35±1.19	45.74±1.98	2.43±0.19	2.67±0.22	4.06±0.63
	*Ranunculus*	-18.97±1.94	10.98±0.84	41.33±0.74	2.26±0.4	-1.06±0.68	5.45±0.45
	Charophytes	-18.38 ±0.86	8.08±1.15	20.6±1.42	0.91±0.09	-1.26±0.3	3.91±0.61

The mean ‰ **±** SD with lipid correction and *δ* ^15^N is specified for each source, as well as the mass fraction (%) and the discrimination factor (*Δ*) for C and N. Food sources were taken from control tanks containing no amphibians or predators. Sample size equaled 6, except for algae *δ* ^15^N (n = 3) and zooplankton *δ* ^15^N (n = 5).

**Fig 4:**
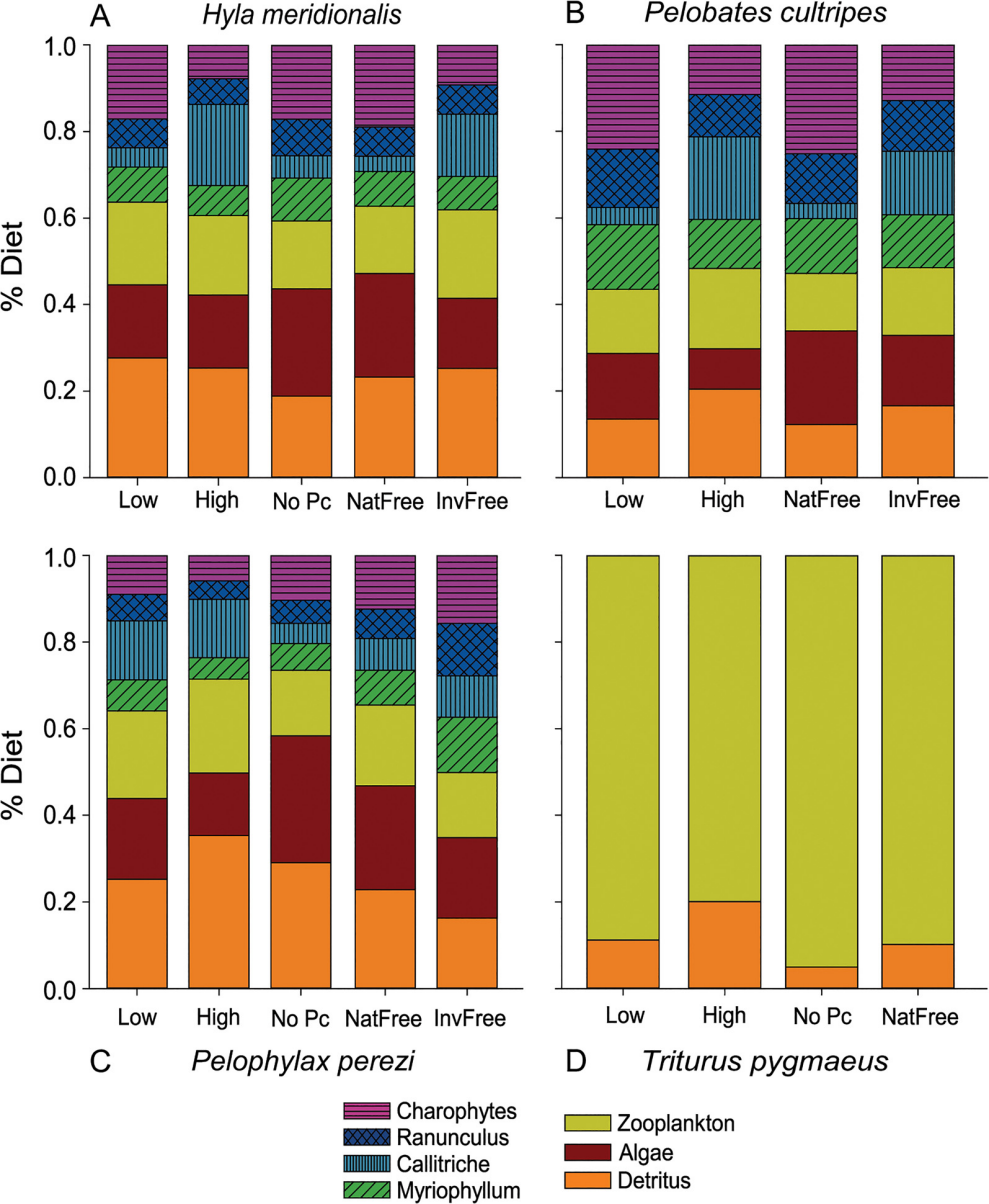
Potential contribution of different food sources to the diet of each amphibian species. Mean estimated proportion of each of the seven potential sources (detritus, algae, zooplankton, and four types of macrophytes) in the diet of three anuran species under five different ecological scenarios: low and high larval density, absence of spadefoot toads (*P*. *cultripes*), presence of free native dytiscid beetles, or presence of invasive red swamp crayfish. In the case of newts (*T*. *pygmaeus*), only two sources were considered since they do not consume macrophytes or algae. Also, no newts survived the presence of free crayfish.

The contribution of the different sources to the diet of amphibians revealed differences among species and among experimental treatments (see Fig 4 and S6–S9 Tables).

Detritus, algae and zooplankton appeared in the diet of all anurans, but their proportions varied among different treatments (Fig 4). Macrophytes (*Ranunculus*, *Callitriche* and *Myriophyllum*) and Charophytes were the main component of the diet in *P*. *cultripes*, with more than 50% of the diet in all treatments (Fig 4B). In the other anuran species, macrophytes were also important food sources, but they were not predominant as in spadefoot toad tadpoles. Only in the case of *P*. *perezi* exposed to free invasive crayfish did macrophytes reach similar importance in the diet (ca. 50%; *InvFree*, Fig 4C). High larval density caused all species (except *H*. *meridionalis*) to increase the proportion of detritus ingested. It was in detriment of the proportion of algae ingested for the species *P*. *cultripes* (Fig 4B) and *P*. *perezi* (Fig 4C). In contrast, at high larval density, the proportion of detritus did not vary for *H*. *meridionalis* (Fig 4A) but it increased the ingestion of one of the macrophyte species (*Callitriche*), which was also the most consumed macrophyte for other anuran species in this treatment (*High*). *Hyla meridionalis* had similar diets when they were at high density as when invasive crayfish were present. However, the diet of *P*. *perezi* varied between these two treatments (*High* and *InvFree*), with a remarkable increase in macrophyte consumption and lower proportion of detritus when they coexisted with invasive free predators (Fig 4C). The presence of crayfish, however, did not seem to affect the general composition of the diet of *P*. *cultripes*, where only the proportion of macrophytes assimilated changed in respect to the absence of predators. In the absence of *P*. *cultripes*, tadpoles of *H*. *meridionalis* slightly increased the proportion of algae in a similar way as occurred when native predators were present (Fig 4A). Although almost negligible, the proportion of macrophytes also increased in comparison to when the entire community was present (Fig 4A). The diet of *Pelophylax perezi*, however, shifted to a lower consumption of macrophytes and a remarkable increase of algae, which in this treatment contributed to diet in a similar proportion as detritus. The presence of native predators increased the proportions of algae in the diets of the three anuran species, which was more evident in the case of *H*. *meridionalis* and *P*. *cultripes*.

Since *T*. *pygmaeus* is a carnivorous species, we only analyzed the variation of zooplankton in relation to detritus as potential food sources. Detritus had a minor contribution to the diet in the absence of *P*. *cultripes* (No Pc), but considerably increased in the high-density treatment (Fig 4D).

## Discussion

### Analyzing the trophic position of amphibian larvae

Differences in *δ* ^15^N and *δ* ^13^C among species and treatments revealed differences among species in their trophic niche, as well as showed substantial flexibility in the use of alternative food sources within species depending on the environmental conditions experienced.

Newts presented high values of *δ* ^15^N, indicating that they fed mostly on animal matter. This is congruent with previous gut content analyses, which showed their carnivorous habits, with a strong preference for cladocerans [39]. Tadpoles are mainly herbivorous in which macrophytes and algae are important food sources, complemented with detritus and zooplankton. This composition coincides with the description of a previous study based on the description of gut contents of the same species [38]. However, in relation to gut analyses, our isotopic studies reveal a higher importance of macrophytes and zooplankton than expected, since they appeared at lower frequencies in gut analyses. Nevertheless, macrophytes and zooplankton are the food sources with higher carbon and nitrogen concentrations (Table 3) and consequently have higher incidence in the assimilation onto tadpole tissues. In fact, these differences illustrate the main advantage of isotopic analyses over gut content analysis for diet determination [35], as it shows the importance of particular resources with high assimilation.

Among tadpoles, spadefoot toads (*P*. *cultripes*) had the most enriched *δ* ^15^N and *δ* ^13^C values, close to the high values of larval newts. Tadpoles of this species caused a great impact on the pond structure, mainly due to their high plant biomass consumption [18]. The high prevalence of macrophytes in their diet thus explains the elevated isotopic signature of this species.

In general, the trophic structure of tadpoles in our study shows a large tadpole species that stands out as the prime macrophyte consumer (*P*. *cultripes*), and two species (*H*. *meridionalis* and *P*. *perezi*) in which other resources have higher importance than macrophytes, but show a wide variation in their proportions related to changes in their availability among treatments. All tadpoles included an additional amount of zooplankton as have been previously reported in their diet [38] and could be the result of scavenging, as suggested by the fact that they often co-vary with detritus consumption.

### Diet shifts under varying conditions

The amphibian species studied did not equally respond to variations in the environmental factors manipulated, like larval density or predator presence. Changes in tadpole feeding may be related to changes in relative abundance of appropriate food items or to the quality of food sources [20]. The inferred changes in diet through stable isotope analysis are consistent with observed changes in the environmental conditions across the experimental treatments, such as altered macrophyte biomass, changing zooplankton composition, and algal abundance.

High larval amphibian density, presence of *P*. *cultripes* tadpoles, and free-roaming invasive crayfish had caused increased nutrient availability in the water column, increased algal abundance, and caused macrophyte depletion [18]. High larval density also increased the proportion of copepods in relation to cladocerans [18], which may be one of the explanations for the slightly different diet composition of larval newts at high density (Fig 4D). The considerable reduction of plant biomass forced herbivorous tadpoles to increase their feeding on detritus, and probably this lack of macrophytes also enhanced the prey capture of larval newts from the detritus. Impoverishment of environmental conditions in the presence of *P*. *cultripes* included plant depletion and shifts in zooplankton composition [18] and these changes were reflected in altered diets of other tadpoles, as we had hypothesized. In general, when *P*. *cultripes* was absent, macrophytes were more abundant and tadpoles showed enriched *δ* ^13^C. However, we observed that the two other tadpole species increased the proportion of algae, but only *H*. *meridionalis* slightly increased the proportion of macrophytes in the diet whereas *P*. *perezi* reduced it in the absence of *P*. *cultripes*. These differences are related to different availability of resources as well as to intrinsic differences in the general food habits between these species. The absence of the strong competitor, *P*. *cultripes*, may result in higher availability of algae and also in lower availability of detritus, as the presence of *P*. *cultripes* increases the proportion of detritus in the bottom of the tanks due to their high consumption of macrophytes. However, under the same availability of macrophytes, one species increased while the other reduced macrophyte consumption. These differences are in agreement with different feeding behavior described for these two species [54] as well as with their diet description based on gut contents, where detritus was one of the most consumed food resources for *P*. *perezi* [38]. In addition, the morphology of the species also indicates that *P*. *perezi* is better adapted to feed at the bottom of the ponds, while *H*. *meridionalis* mainly feeds on the water column [38], where they probably also consume macrophytes as a complementary alternative resource. Newts also increased the proportion of zooplankton in their diet when *P*. *cultripes* was absent, showing higher *δ* ^13^C but substantially lower *δ* ^15^N values. This may be attributed to the observed differences in species composition of zooplankton [18], since cladocerans increased in relative abundance with respect to copepods, and cladocerans typically show lower *δ* ^15^N values than copepods [55]. Spadefoot toad larvae can thus be considered a strong competitor that markedly reduces the availability of resources that would otherwise be readily consumed by the rest of the amphibian guild. We expected native predators to cause an increase in the trophic level of surviving amphibians, since they would have access to more abundant and diverse food sources (including those with higher *δ* ^15^N values). Our results do not support our prediction since released competition via predation did not significantly alter the diet of amphibian larvae.

In accordance with our expectations, freely roaming crayfish produced a considerable change in the trophic status of tadpoles, with *H*. *meridionalis* and *P*. *cultripes* showing a slight decrease in *δ* ^13^C values whereas *P*. *perezi* experienced a similar increase as when *P*. *cultripes* was absent from the tanks. Invasive crayfish have a high disruptive potential of the trophic webs of aquatic systems [56,57]. In our mesocosms, crayfish had a similar effect to that of high density of tadpoles, but had different consequences in different species. Only the diet composition of *H*. *meridionalis* was similar in both treatments. Also, the presence of crayfish did not affect the diet of *P*. *cultripes*, which was in general similar to that in the low-density treatment. The composition of the macrophytes, however, was more similar to the high-density treatment. Crayfish largely contributed to deplete plant biomass and impoverished water quality [18], presumably forcing tadpoles to increase their ingestion of sediments or to feed on C-depleted resources. This also happened in the presence of *P*. *cultripes*, mainly at high densities. However, tadpoles of *P*. *perezi* did not modify their diet in the same way as in high density; instead they increased the proportion of plants in their diet in the presence of crayfish, considerably increasing their *δ* ^15^N. Crayfish commonly uproot or cut plants to favor the growth of bacteria and fungi that complement their diet [58]. This feeding behavior can increase the plant material included in sediments, favoring their consumption by detritivorous bottom dwellers such as *P*. *perezi*. Therefore, crayfish act not only as predators of amphibian larvae [59–61] but also compete with some of them [43] for resources like algae and macrophytes. They may in turn favor more detritivorous species. These effects were not found when crayfish were caged, and the isotopic values of tadpoles were similar to those in the absence of predators. Nevertheless, larval newts showed a decrease in *δ* ^15^N with caged crayfish, which was likely due to the reduction in copepod abundance in favor of cladocerans caused by caged crayfish [18].

## Conclusion

The combination of ecological experiments and stable isotope analyses offers a powerful approach to test the effect of environmental changes on the trophic adjustment of interacting species and its consequences for food web structure. Our results show that the anuran species studied are not specialists of specific types of food but rather opportunistic herbivores and/or detritivores. However, despite some degree of niche partitioning, the species studied were at the same time capable of exploiting a variable proportion of different food resources (algae, zooplankton, macrophytes, detritus), depending on the ecological scenarios. Thus, which food source predominates in each species’ diet is largely conditioned by both intraspecific and interspecific competition, whereas we found little evidence for non-consumptive, trait-mediated predator effects. Changes in diet due to different ecological interactions have direct or indirect consequences on the local structure of an ecosystem, as the food web interactions are also likely to be altered. The observed shifts in the amphibians’ diet in response to environmental changes demonstrate that amphibians are greatly affected by intraspecific density, interspecific competition, and invasive species. This ability of amphibian larvae to alter their trophic niche depending on the ecological scenario may be determinant in their capacity for exploiting widely divergent or fluctuating conditions in the large array of aquatic systems they inhabit. Such shifts in the trophic niche of larval amphibians may strongly affect the structure and dynamics of the food web of temporary ponds, especially if such changes occur at a fast rate due to the introduction of invasive species or other factors causing rapid declines of amphibian populations.

## Supporting Information

S1 Table Initial total body length of the amphibian larvae and final total body length of the amphibian larvae or metamorphs of the species *Hyla meridionalis* included in each of the experimental treatment of the experiment. (DOCX)

S2 Table Initial total body length of the amphibian larvae and final total body length of the amphibian larvae or metamorphs of the species *Pelobates cultripes* included in each of the experimental treatment of the experiment. (DOCX)

S3 Table Initial total body length of the amphibian larvae and final total body length of the amphibian larvae or metamorphs of the species *Pelophylax perezi* included in each of the experimental treatment of the experiment. (DOCX)

S4 Table Initial total body length of the amphibian larvae and final total body length of the amphibian larvae or metamorphs of the species *Triturus pygmaeus* included in each of the experimental treatment of the experiment. (DOCX)

S5 Table Stable isotopic values of carbon and nitrogen (mean ± SE) of the four amphibian species included in the experiment. (DOCX)

S6 Table Potential food sources contributing to the diet of the larvae of the species *Hyla meridionalis*. (DOCX)

S7 Table Potential food sources contributing to the diet of the larvae of the species *Pelobates cultripes*. (DOCX)

S8 Table Potential food sources contributing to the diet of the larvae of the species *Pelophylax perezi*. (DOCX)

S9 Table Potential food sources contributing to the diet of the larvae of the species *Triturus pygmaeus*. (DOCX)

S10 Table Isotopic signatures of all the samples included in the experiment. For both sources and amphibians we show the Delta 13C x 1000 (corrected for the amphibians following the equation from Caut et al. 2013), elemmental composition of carbon (Mass Fraction Cx100 or MASSC100), Delta 15N x 1000, and elemmental composition of nitrogen (Mass Fraction Nx100, or MASSN15). For the sources (first tab), we also show the division of Mass Fraction C and Mass Fraction N (C/N), the discrimation factor for *δ* ^13^C (following the equation in Caut et al 2013; *Δ* ^13^C = -0.35* *δ* ^13^C -7.7) and the discrimation factor for *δ* ^15^N (following the equation in Caut et al 2013; *Δ* ^15^N = 0.53* *δ* ^15^N -0.37) of tadpoles for the various resources available. We also show the enriched13C and *δ* ^15^N, after taking into account the discrimation factors for each resource. For the amphibians, we show the treatment for each sampled individual (more details in the text), the type (met if metamorphic, and tad if tadpole), the date (in the year 2011) and the weight in grames. Each tab includes the data for each amphibian species included in the experiment (*Hyla meridionalis*, *Pelobates cultripes*, *Pelophylax perezi*, *Triturus pygmaeus)*.
(XLSX)
