# Supplementary material for: Stable Isotopes Reveal Trophic Partitioning and Trophic Plasticity of a Larval Amphibian Guild
Source: PLoS One. 2015 Jun 19;10(6):e0130897. doi: 10.1371/journal.pone.0130897 (PMC4474902; doi:10.1371/journal.pone.0130897)
Supplement: S1 Table — (DOCX) [file pone.0130897.s001.docx]

| ***Hyla meridionalis*** | | | | | |
| --- | --- | --- | --- | --- | --- |
|  |  | Experiment | | Isotopic analysis | |
| **Treatment** | **Initial TL** | **Final TL (tadpoles)** | **Final TL (metamorphs)** | **Final TL (tadpoles)** | **Final TL (metamorphs)** |
| **Low** | 23.06 ± 0.61  (n=120) | 34.17 ± 1.74 (n=3) | 14.11 ± 0.31 (n=34) | (n=0) | 12.96 ± 0.18 (n=10) |
| **High** | 24.21 ± 0.36  (n=360) | 25.13 ± 0.98 (n=8) | 14.03 ± 0.29 (n=22) | 24.07±1.37 (n=5) | 12.3 (n=1) |
| **No Pc** | 23.96 ± 0.6  (n=120) | (n=0) | 15.22 ± 0.16 (n=65) | (n=0) | 15.33 ± 0.44 (n=11) |
| **Nat Caged** | 24.75 ± 0.66  (n=120) | 30.66 ± 2.7 (n=3) | 13.96 ± 0.26 (n=39) | (n=0) | 12.96 ± 0.46 (n=12) |
| **Nat Free** | 23.27 ± 0.54  (n=120) | (n=1) | 14.27 ± 0.28 (n=24) | (n=0) | 14.04 ± 0.38 (n=9) |
| **Inv Caged** | 23.78 ± 0.64  (n=120) | 32.3 (n=1) | 14 ± 0.19 (n=50) | (n=0) | 13.05 ± 0.4 (n=11) |
| **Inv Free** | 23.26 ± 0.6  (n=120) | (n=0) | 14.51 ± 0.23 (n=35) | (n=0) | 14.03 ± 0.72 (n=7) |

**S1 Table. Initial total body length of the amphibian larvae and final total body length of the amphibian larvae or metamorphs of the species *H. meridionalis* included in each of the experimental treatment of the experiment.**

Length is expressed in mm (TL, mean ± SE). Number of individuals is specified in brackets and for this species the initial number was 10 individuals per tank in low density. We specify the final TL and number of all individuals in the experiment, and also for the individuals used only in the isotopic analysis.
