# Supplementary material for: Stable Isotopes Reveal Trophic Partitioning and Trophic Plasticity of a Larval Amphibian Guild
Source: PLoS One. 2015 Jun 19;10(6):e0130897. doi: 10.1371/journal.pone.0130897 (PMC4474902; doi:10.1371/journal.pone.0130897)
Supplement: S3 Table — (DOCX) [file pone.0130897.s003.docx]

| ***Pelophylax perezi*** | | | | | |
| --- | --- | --- | --- | --- | --- |
|  |  | Experiment | | Isotopic analysis | |
| **Treatment** | **Initial TL** | **Final TL (tadpoles)** | **Final TL (metamorphs)** | **Final TL (tadpoles)** | **Final TL (metamorphs)** |
| **Low** | 23.65 ± 2.41 (n=36) | 36.74 ± 2.62 (n=13) | 18.5 (n=2) | 34.06 ± 4.03 (n=7) | 18.5 (n=1) |
| **High** | 22.02 ± 1.26 (n=108) | 30.67 ± 1.26 (n=31) | 16.2 ± 0.65 (n=6) | 28.82 ± 2.43 (n=9) | 16.15 ± 1.45 (n=2) |
| **No Pc** | 22.31 ± 2.17 (n=36) | 45.65 ± 1.93 (n=20) | 18.9 ± 0.45 (n=4) | 47.12 ± 2.73 (n=9) | 18.4 ± 1.2 (n=2) |
| **Nat Caged** | 21.77 ± 2 (n=36) | 36.13 ± 2.73 (n=12) | 17.63 ± 1.39 (n=3) | 34.05 ± 3.15 (n=8) | 17.63 ± 1.39 (n=3) |
| **Nat Free** | 21.78 ± 2.18 (n=36) | 47.65 ± 2.83 (n=14) | 19.03 ± 0.38 (n=4) | 43.19 ± 4.34 (n=7) | 19.03 ± 0.38 (n=3) |
| **Inv Caged** | 22.65 ± 2.25 (n=36) | 40.62 ± 2.54 (n=16) | 18.1 ± 0.62 (n=4) | 41.27 ± 2.93 (n=8) | 18.1 ± 0.62 (n=3) |
| **Inv Free** | 22.55 ± 2.33 (n=36) | 38.76 ± 2.51 (n=11) | n=1 (no measures) | 39.75 ± 3.7 (n=7) | (n=0) |

**S3 Table. Initial total body length of the amphibian larvae and final total body length of the amphibian larvae or metamorphs of the species *P. perezi* included in each of the experimental treatment of the experiment.** Length is expressed in mm (TL, mean ± SE). Number of individuals is specified in brackets and for this species the initial number was 3 individuals per tank in low density. We specify the final TL and number of all individuals in the experiment and for the individuals used in the isotopic analysis.
