# Supplementary material for: Stable Isotopes Reveal Trophic Partitioning and Trophic Plasticity of a Larval Amphibian Guild
Source: PLoS One. 2015 Jun 19;10(6):e0130897. doi: 10.1371/journal.pone.0130897 (PMC4474902; doi:10.1371/journal.pone.0130897)
Supplement: S4 Table — (DOCX) [file pone.0130897.s004.docx]

| ***Triturus pygmaeus*** | | | | | |
| --- | --- | --- | --- | --- | --- |
| **Species** |  | Experiment | | Isotopic analysis | |
| **Treatment** | **Initial TL** | **Final TL (tadpoles)** | **Final TL (metamorphs)** | **Final TL (larvae)** | **Final TL (metamorphs)** |
| **Low** | 30.6 ± 0.6 (n=36) | 52.5 ± 0.93 (n=19) | 53.45 ± 0.95 (n=2) | 52.54 ± 1.86 (n=7) | 53.45 ± 0.95 (n=2) |
| **High** | 31.67 ± 0.42 (n=108) | 44.11 ± 0.54 (n=79) | 52.4 (n=1) | 49.14 ± 1.46 (n=9) | 52.4 (n=1) |
| **No Pc** | 31.66 ± 0.71 (n=36) | 50.95 ± 0.92 (n=18) | 54.11 ± 0.88 (n=8) | 53.45 ± 1.42 (n=12) | 55.1 ± 0.5 (n=2) |
| **Nat Caged** | 32.81 ± 0.58 (n=36) | 53.05 ± 0.84 (n=23) | 55.5 (n=1) | 53.67 ± 1.64 (n=10) | 55.5 (n=1) |
| **Nat Free** | 32.02 ± 0.89 (n=36) | 54 ± 1.24 (n=20) | 49.15 ± 2.05 (n=2) | 56.38 ± 2.55 (n=7) | 49.15 ± 2.05 (n=2) |
| **Inv Caged** | 31.8 ± 0.88 (n=36) | 52.97 ± 0.67 (n=29) | 51.42 ± 0.41 (n=4) | 54.81 ± 1.28 (n=10) | 51.3 ± 0.56 (n=3) |
| **Inv Free** | 32.56 ± 0.8 (n=36) | (n=0) | (n=0) | (n=0) | (n=0) |

**S4 Table. Initial total body length of the amphibian larvae and final total body length of the amphibian larvae or metamorphs of the species *T. pygmaeus* included in each of the experimental treatment of the experiment.** Length is expressed in mm (TL, mean ± SE). Number of individuals is specified in brackets and for this species the initial number was 3 individuals per tank in low density. We specify the final TL and number of all individuals in the experiment and for the individuals used in the isotopic analysis.
