# Supplementary material for: Stable Isotopes Reveal Trophic Partitioning and Trophic Plasticity of a Larval Amphibian Guild
Source: PLoS One. 2015 Jun 19;10(6):e0130897. doi: 10.1371/journal.pone.0130897 (PMC4474902; doi:10.1371/journal.pone.0130897)
Supplement: S5 Table — (DOCX) [file pone.0130897.s005.docx]

| **Treatment** | ***δ*^13^C ‰** | | | | ***δ*^15^N ‰** | | | |
| --- | --- | --- | --- | --- | --- | --- | --- | --- |
|  | *H. meridionalis* | *P. cultripes* | *P. perezi* | *T. pygmaeus* | *H. meridionalis* | *P. cultripes* | *P. perezi* | *T. pygmaeus* |
| **Low** | -22.4 ± 0.27 | -21.52 ± 0.23 | -24.08 ± 0.87 | -22.5 ± 0.52 | 7.66 ± 0.39 | 10.22 ± 0.24 | 7.83 ± 0.49 | 10.51 ± 0.43 |
|  | (-24.14, -20.93) | (-22.88, -19.6) | (-29.19, -21.66) | (-24.85, -19.93) | (5.69, 9.12) | (8.37, 11.58) | (6.15, 10.04) | (9.18, 13.4) |
|  | n=10 | n=16 | n=8 | n=9 | n=10 | n=16 | n=8 | n=9 |
| **High** | -24.45 ± 0.67 | -23.26 ± 0.18 | -27.88 ± 0.85 | -24.97 ± 0.35 | 8.41 ± 0.29 | 9.56 ± 0.19 | 6.86 ± 0.32 | 10.36 ± 0.19 |
|  | (-26.67, -22.09) | (-25.21, -22.02) | (-31.19, -24.74) | (-26.92, -22.8) | (7.83, 9.49) | (7.4, 10.78) | (5.32, 8.55) | (9.37, 11.33) |
|  | n=6 | n=18 | n=11 | n=10 | n=7 | n=18 | n=11 | n=10 |
| **No Pc** | -21.61 ± 0.50 |  | -20.85 ± 0.99 | -21.12 ± 0.24 | 8.23 ± 0.50 |  | 4.42 ± 0.56 | 9.15 ± 0.25 |
|  | (-24.44, -19.39) |  | (-26.74, -17.26) | (-22.42, -19.35) | (4.23, 10.35) |  | (2.08, 7.47) | (7.79, 10.7) |
|  | n=11 |  | n=11 | n=14 | n=11 |  | n=11 | n=14 |
| **Nat Caged** | -22.02 ± 0.35 | -21.74 ± 0.32 | -24.58 ± 0.88 | -23.25 ± 0.43 | 8.13 ± 0.33 | 10.34 ± 0.21 | 7.69 ± 0.29 | 10.34 ± 0.3 |
|  | (-24, -20.11) | (-24.26, -19.12) | (-28.68, -18.38) | (-25.79, -21.23) | (6.27, 10.18) | (9.21, 12.01) | (6.34, 9.62) | (8.48, 12.01) |
|  | n=12 | n=14 | n=11 | n=11 | n=11 | n=14 | n=11 | n=11 |
| **Nat Free** | -21.41 ± 0.27 | -21.26 ± 0.32 | -22.29 ± 0.75 | -21.59 ± 0.59 | 7.48 ± 0.39 | 9.75 ± 0.24 | 7.78 ± 0.46 | 9.89 ± 0.32 |
|  | (-22.5, -20.27) | (-24.78, -19.24) | (-27.86, -20.03) | (-24.49, -19.79) | (4.81, 8.72) | (8.49, 11.62) | (5.89, 10.83) | (8.75, 11.38) |
|  | n=9 | n=16 | n=10 | n=9 | n=9 | n=16 | n=10 | n=9 |
| **Inv Caged** | -22.33 ± 0.23 | -21.36 ± 0.28 | -24.25 ± 0.47 | -22.41 ± 0.27 | 8.05 ± 0.27 | 10.07 ± 0.36 | 7.82 ± 0.48 | 9.71 ± 0.21 |
|  | (-23.45, -20.98) | (-23.97, -19.74) | (-26.69, -21.99) | (-23.7, -20.25) | (6.75, 9.29) | (7.65, 12.38) | (6.18, 12.07) | (8.34, 11.09) |
|  | n=11 | n=16 | n=11 | n=13 | n=11 | n=16 | n=11 | n=13 |
| **Inv Free** | -23.59 ± 0.42 | -22.67 ± 0.58 | -21.09 ± 0.91 | - | 7.93± 0.55 | 9.58 ± 0.8 | 9.16 ± 0.65 | - |
|  | (-24.95, -22.18) | (-25.53, -21.06) | (-25.72, -19.06) | - | (6.22, 9.69) | (6.5, 12.56) | (5.39, 10.63) | - |
|  | n=7 | n=7 | n=7 | n=0 | n=7 | n=7 | n=7 | n=0 |

**S5 Table**. Stable isotopic values of carbon and nitrogen (mean ± 1 SE) of the different amphibian species included in the experiment, shown by treatments. The range of isotopic values (min – max, in parentheses) and the number of individuals per treatment (n) is also shown. Carbon values are corrected for lipids to account for variation (mean C:N = 3.51), following the equation for amphibian tadpoles in Caut et al. [1]

1. Caut S, Angulo E, Díaz-Paniagua C, Gomez-Mestre I (2013) Plastic changes in tadpole trophic ecology revealed by stable isotope analysis. Oecologia 173: 95–105.
