# Supplementary material for: Stable Isotopes Reveal Trophic Partitioning and Trophic Plasticity of a Larval Amphibian Guild
Source: PLoS One. 2015 Jun 19;10(6):e0130897. doi: 10.1371/journal.pone.0130897 (PMC4474902; doi:10.1371/journal.pone.0130897)
Supplement: S6 Table — (DOCX) [file pone.0130897.s006.docx]

**S6 Table.** Percentage (mean ± SD) and 95% confidence interval of each potential food source contributing to the diet of the larvae of the species *H. meridionalis* (Mediterranean treefrog) included in the experiment in the density treatments and in the predator-free treatments (both dityscid larvae -*NatFree*- and red swamp crayfish –*InvFree*-). Values reported resulted as output from SIAR models.

| ***Hyla meridionalis*** | | | | | | | | | | |
| --- | --- | --- | --- | --- | --- | --- | --- | --- | --- | --- |
| **Treatment** | **Low** | | **High** | | **No Pc** | | **NatFree** | | **InvFree** | |
| Source | % | 95% | % | 95% | % | 95% | % | 95% | % | 95% |
| **Detritus** | 0.28 ± 0.09 | 0.11-0.44 | 0.25 ± 0.09 | 0.09-0.43 | 0.19 ± 0.08 | 0.015-0.34 | 0.23 ± 0.09 | 0.05-0.4 | 0.25 ± 0.09 | 0.08-0.43 |
| **Algae** | 0.17 ± 0.07 | 0.018-0.3 | 0.17 ± 0.09 | 0-0.33 | 0.25 ± 0.09 | 0.06-0.43 | 0.24 ± 0.09 | 0.06-0.41 | 0.16 ± 0.08 | 0.003-0.3 |
| **Zooplankton** | 0.19 ± 0.11 | 0-0.37 | 0.18± 0.09 | 0.002-0.34 | 0.16 ± 0.09 | 0-0.32 | 0.16 ± 0.1 | 0-0.33 | 0.21 ± 0.11 | 0.001-0.4 |
| ***Myriophyllum*** | 0.08 ± 0.06 | 0-0.2 | 0.07 ± 0.05 | 0-0.18 | 0.1 ± 0.07 | 0-0.23 | 0.08 ± 0.06 | 0-0.2 | 0.08 ± 0.06 | 0-0.19 |
| ***Callitriche*** | 0.05 ± 0.04 | 0-0.12 | 0.19 ± 0.08 | 0.018-0.33 | 0.05 ± 0.04 | 0-0.14 | 0.04 ± 0.03 | 0-0.1 | 0.14 ± 0.07 | 0.006-0.27 |
| ***Ranunculus*** | 0.07 ± 0.05 | 0-0.17 | 0.06 ± 0.05 | 0-0.15 | 0.08 ± 0.06 | 0-0.21 | 0.07 ± 0.06 | 0-0.18 | 0.07 ± 0.05 | 0-0.17 |
| **Charophytes** | 0.17 ± 0.09 | 0.005-0.33 | 0.08 ± 0.06 | 0-0.2 | 0.17 ± 0.09 | 0.001-0.33 | 0.19 ± 0.1 | 0.0022-0.36 | 0.09 ± 0.07 | 0-0.22 |
