# Supplementary material for: Stable Isotopes Reveal Trophic Partitioning and Trophic Plasticity of a Larval Amphibian Guild
Source: PLoS One. 2015 Jun 19;10(6):e0130897. doi: 10.1371/journal.pone.0130897 (PMC4474902; doi:10.1371/journal.pone.0130897)
Supplement: S8 Table — (DOCX) [file pone.0130897.s008.docx]

**S8 Table.** Percentage (mean ± SD) and 95% confidence interval of each potential food source contributing to the diet of the larvae of the species *P. perezi* (Iberian green frog) included in the experiment in the density treatments and in the predator-free treatments (both dityscid larvae -*NatFree*- and red swamp crayfish –*InvFree*-). Values reported resulted as output from SIAR models.

| ***Pelophylax perezi*** | | | | | | | | | | |
| --- | --- | --- | --- | --- | --- | --- | --- | --- | --- | --- |
| **Treatment** | **Low** | | **High** | | **No Pc** | | **NatFree** | | **InvFree** | |
| Source | % | 95% | % | 95% | % | 95% | % | 95% | % | 95% |
| **Detritus** | 0.25 ± 0.09 | 0.066-0.44 | 0.35 ± 0.11 | 0.14-0.57 | 0.29 ± 0.13 | 0.025-0.55 | 0.23 ± 0.09 | 0.034-0.4 | 0.16 ± 0.08 | 0.005-0.3 |
| **Algae** | 0.19 ± 0.1 | 0-0.36 | 0.14 ± 0.11 | 0-0.34 | 0.29 ± 0.14 | 0.022-0.57 | 0.24 ± 0.11 | 0.017-0.44 | 0.18 ± 0.09 | 0.004-0.34 |
| **Zooplankton** | 0.2 ± 0.11 | 0-0.4 | 0.22 ± 0.13 | 0-0.45 | 0.15 ± 0.1 | 0-0.34 | 0.19 ± 0.11 | 0-0.37 | 0.15 ± 0.09 | 0-0.29 |
| ***Myriophyllum*** | 0.07 ± 0.06 | 0-0.18 | 0.05 ± 0.04 | 0-0.13 | 0.06 ± 0.06 | 0-0.18 | 0.08 ± 0.06 | 0-0.2 | 0.13 ± 0.08 | 0-0.26 |
| ***Callitriche*** | 0.14 ± 0.08 | 0-0.28 | 0.14 ± 0.08 | 0-0.28 | 0.05 ± 0.04 | 0-0.14 | 0.07 ± 0.06 | 0-0.19 | 0.1 ± 0.07 | 0-0.23 |
| ***Ranunculus*** | 0.06 ± 0.05 | 0-0.16 | 0.04 ± 0.04 | 0-0.11 | 0.05 ± 0.05 | 0-0.16 | 0.07 ± 0.05 | 0-0.17 | 0.12 ± 0.08 | 0-0.25 |
| **Charophytes** | 0.09 ± 0.07 | 0-0.22 | 0.06 ± 0.05 | 0-0.16 | 0.1 ± 0.08 | 0-0.26 | 0.12 ± 0.08 | 0-0.27 | 0.16 ± 0.09 | 0-0.3 |
