# Supplementary material for: Stable Isotopes Reveal Trophic Partitioning and Trophic Plasticity of a Larval Amphibian Guild
Source: PLoS One. 2015 Jun 19;10(6):e0130897. doi: 10.1371/journal.pone.0130897 (PMC4474902; doi:10.1371/journal.pone.0130897)
Supplement: S9 Table — (DOCX) [file pone.0130897.s009.docx]

**S9 Table.** Percentage (mean ± SD) and 95% confidence interval of each potential food source contributing to the diet of the larvae of the species *T. pigmaeus* (pigmy marbled newt) included in the experiment in the density treatments and in the predator-free treatments (dityscid larvae -*NatFree*). Values reported resulted as output from SIAR models. For this species we included only detritus and zooplankton as potential food sources, and excluded treatments for which there were no survivors (*InvFree*).

| ***Triturus pygmaeus*** | | | | | | | | |
| --- | --- | --- | --- | --- | --- | --- | --- | --- |
| **Treatment** | **Low** | | **High** | | **NoPc** | | **NatFree** | |
| Source | % | 95% | % | 95% | % | 95% | % | 95% |
| **Detritus** | 0.11 ± 0.1 | 0-0.36 | 0.2 ± 0.16 | 0-0.52 | 0.05 ± 0.05 | 0-0.15 | 0.1 ± 0.1 | 0-0.33 |
| **Zooplankton** | 0.89 ± 0.1 | 0.64-1 | 0.8 ± 0.16 | 0.48-1 | 0.95 ± 0.05 | 0.85-1 | 0.9 ± 0.1 | 0.67-1 |
